# Cell Structure and Dynamics of Galactomannan Secretion in *Caesalpinia pulcherrima* (Leguminosae) Endosperm

**DOI:** 10.3390/plants15010076

**Published:** 2025-12-26

**Authors:** Victor Bonifácio-Leite, Élder Antônio Sousa Paiva, Denise M. T. Oliveira

**Affiliations:** Departamento de Botânica, Instituto de Ciências Biológicas, Universidade Federal de Minas Gerais, Belo Horizonte 31270-901, Minas Gerais, Brazil; victorbonifacioleite@gmail.com (V.B.-L.); denisemtoliveira@gmail.com (D.M.T.O.)

**Keywords:** storage polysaccharides, endosperm ultrastructure, leguminous seeds, secretory cell, seed reserve

## Abstract

Galactomannans are a typical reserve polysaccharide in the endosperm of leguminous seeds; they turn the endosperm hard when dry and gelatinous and swollen when hydrated. Although galactomannans of several species have been biochemically characterized, little is known about their deposition within the endosperm. This study aimed to clarify how polysaccharides, galactomanans according to the literature, are produced and stored in the endosperm of *Caesalpinia pulcherrima* seeds by describing its structural and ultrastructural features throughout development. Samples of seeds at different developmental stages were collected and processed for study under light and electron microscopy. During development, the endosperm of *C. pulcherrima* undergoes substantial anatomical modifications associated with cellular cycles of polysaccharide release that gradually accumulates in the intercellular spaces. Endosperm cells exhibit an active Golgi apparatus with intense polysaccharide production, confirming their secretory function. In the mature endosperm, polysaccharides are stored in periplasmic and intercellular spaces rather than in thickened cell walls, as previously reported for other Leguminosae. By showing that galactomannans accumulate in periplasmic and intercellular spaces rather than in cell walls, our findings expand current understanding of endosperm diversity in Leguminosae and provide a foundation for future comparative studies on galactomannan synthesis and deposition across the family.

## 1. Introduction

The amount of endosperm in Leguminosae seeds is highly diverse. It varies from a trace confined to the radicular region to encasing the embryo with a thickness comparable to the cotyledons [1]. The endosperm is usually hard and vitreous in dry, mature seeds, but it becomes gelatinous, and volume increases when soaked [1]. This change in consistency is attributed to the hydrophilic properties of galactomannans present in the endosperm of most leguminous species [2]. Among the many legumes that exhibit this feature, *Caesalpinia pulcherrima* (L.) Sw. is a traditional medicinal legume originally from Central America and widely distributed in tropical and subtropical regions [3,4,5]. It has been increasingly studied for its pharmacological properties ([6] and references therein). The galactomannans from its seeds have likewise been investigated as an alternative to commercial gums, functioning as viscosity modifiers and thickening agents in the food industry [4,7]. As a result, several studies have focused on characterizing biochemical and physicochemical properties of galactomannans from the endosperm [4,8,9,10,11,12]. These polysaccharides account for around 30% of the total mature seed weight and exhibit an M:G ratio ranging from 1.9 to 3.0 [4,8,9,11]. Additionally, pure mannans have been detected in the endosperm of seeds during maturation, and it was proposed that mannans, rather than cellulose, are deposited in endosperm cell walls as structural polysaccharides during seed development [13]. Although the galactomannans of *C. pulcherrima* have been widely bio- and physicochemically characterized, the structure and ultrastructure of the endosperm, especially the cellular mechanisms involved in the synthesis of these polysaccharides, remain poorly understood.

The endosperm performs critical roles during seed development, germination, and early seedling growth, including embryo nourishment, signalling, mechanical protection, and dormancy maintenance [14]. The endosperm is best known for its storage function in which reserve substances are accumulated during seed development and later mobilized to support post-germinative seedling growth [15]. These substances are usually storage proteins, oils, and carbohydrates, the latter being mainly as starch [16]. In addition to the common reserves, species from several families, such as Annonaceae, Arecaceae, Leguminosae, and Iridaceae, are known to accumulate storage polysaccharides other than starch in the endosperm, which serve as the main carbohydrate reserves in the seed [2]. These polysaccharides, commonly referred to as cell wall storage polysaccharides (CWSP), comprise three major groups: mannans, xyloglucans, and galactans [2,14,17]. These CWSPs are synthesized in the Golgi apparatus, deposited in the endosperm cell during seed development, and are later mobilized during post-germinative growth [14,18]. However, it is important to emphasize that structural polysaccharides may accumulate outside the cell wall, independent of the wall matrix [19,20], or be released as exudates, such as gums and mucilages [21].

The mannan family comprises one of the largest groups of CWSP present in seeds [8,9]. This family includes polysaccharides with a main chain of D-mannose residues linked by β-(1,4) bonds. The D-mannose residues may be variably substituted with D-galactose units through α-(1,6)-glycosidic linkages, resulting in polymers with different mannose-to-galactose ratios (M:G ratio) [22,23]. Polysaccharides with mannose content above 90% are usually regarded as pure mannans; otherwise, those with lower mannan proportions are considered galactomannans [24]. When the main polymer chains of pure mannans and galactomannans also include D-glucose residues, the polysaccharides are regarded as glucomannans and galactoglucomannans, respectively [25,26]. In addition to their storage role, there appears to be a structure–function relationship that determines additional roles of mannans in seeds. As galactose branching approaches zero (pure mannans), their function tends to be more related to hardness due to the presence of water-insoluble crystalline mannans in the endosperm cell walls, as observed in palms and non-legume species [9,14]. In contrast, galactomannans with increasingly higher degrees of galactose substitution are hydrophilic and become gelatinous after a period of imbibition [2]. Due to this feature, the endosperm can act as a “water buffer”, and its role in regulating water relations during germination has been proposed [2,9,27].

Galactomannans, sometimes referred to as “gums”, are neutral polysaccharides widely present in the endosperm of leguminous seeds [2,26]. Species such as *Tara spinosa* (Molina) Britton & Rose (tara, synonym of *Caesalpinia spinosa* (Molina) Kuntze), *Ceratonia siliqua* L. (carob or locust bean), *Cyamopsis tetragonoloba* Taub. (guar) are of considerable economic relevance, and have long been exploited as sources of mannans for industrial applications in the food, paper, pharmaceutical, and cosmetic sectors [2,26,28]. Accordingly, biochemical and physicochemical analyses have been conducted on the galactomannans of several leguminous seeds, and a few have been considered as potential alternative sources to commercial gums [9,10]. Within Leguminosae, these polysaccharides show considerable compositional variation: the M:G ratio ranges from 1.0 to 5.5 [9], and galactomannans may account for 15 to 38% of the seed dry weight [28].

Studies on the structural and ultrastructural aspects related to galactomannan deposition and mobilization are scarce and limited to only a few leguminous species [9,19,20,22,23,29,30,31], which do not represent the diversity of the family that counts with around 19,500 species [32]. The endosperm of some species, such as *C. tetragonoloba* [33], *Medicago sativa* L., *Trigonella foenum-graecum* L., and *Trifolium incarnatum* L. [29], is mainly composed of non-living storage cells, whose lumen is almost filled with galactomannans [29,33]. Adjacent to the seed coat, the endosperm of these species exhibits an aleurone layer, a dermal uniseriate layer (except for *C. tetragonoloba*, which is multiseriate) of living cells responsible for releasing the enzymes that digest the galactomannans filling the lumens of the storage cells [29,34,35]. In contrast, *C. siliqua* lacks an aleurone layer; all endosperm cells are living, with galactomannans forming a layer of variable thickness beneath the cell walls, and are involved in producing the enzymes responsible for galactomannan digestion [31]. *Cercis siliquastrum* L. also lacks an aleurone layer; the endosperm cells are living, and the polysaccharides are deposited as thickenings of the cell walls and within the intercellular spaces [19,20]. In this species, galactoglucomannans rather than galactomannans are deposited in the endosperm [36]. In *Sesbania virgata* (Cav.) Poir. (formerly *Sesbania marginata* Benth.), an aleurone layer occurs together with living endosperm cells containing galactomannan in their cell walls [23]. Although the few previous reports reveal substantial diversity in the cellular and anatomical patterns related to galactomannan deposition within the endosperm, observations on the ultrastructural aspects of the cells involved in galactomannan synthesis are available only for *T. foenum-graecum* [37].

Given their industrial applications, several authors have elucidated the biochemical composition and physicochemical properties of galactomannans present in leguminous seeds. However, the anatomical and ultrastructural aspects of galactomannan synthesis and deposition remain poorly understood. Therefore, the objective of this study is to explain how galactomannans are produced and stored in the endosperm of *Caesalpinia pulcherrima* seeds by describing their structural and ultrastructural features throughout development.

## 2. Results

### 2.1. Endosperm Structure

**Figure 1:**
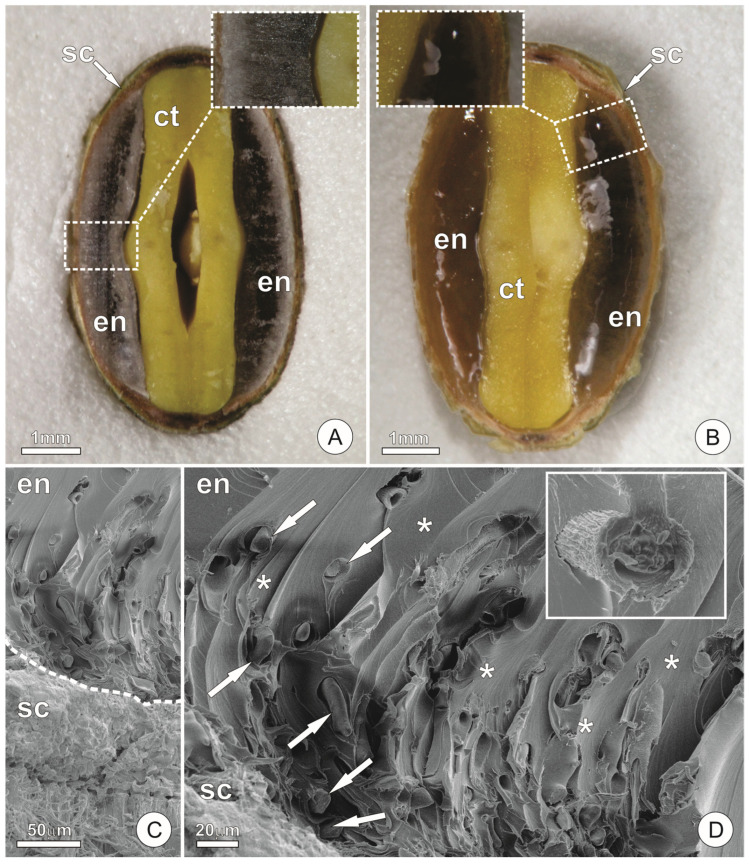
General structure of the endosperm of *Caesalpinia pulcherrima*. (**A**) Median cross-section of a dry mature seed, highlighting the aspect of the dehydrated endosperm. (**B**) Median cross-section of a mature seed in (**A**) after soaking in water, showing the endosperm at the beginning of the imbibition and gelatinization process. (**C**,**D**) Endosperm and seed coat observed under scanning electron microscopy. In **C**, the white dashed line indicates the boundary between the endosperm and the seed coat. (**D**) Endosperm cells (arrows) embedded in the matrix formed by the exudates occupying the intercellular spaces (asterisks). In the insert, see a cell in detail. (ct = cotyledons; en = endosperm; sc = seed coat).

In dry mature seeds (stage 4), the endosperm of *C. pulcherrima* is hard and exhibits a vitreous appearance (Figure 1A). The endosperm surrounds the embryo and, in transverse section, shows approximately the same thickness as the cotyledons (Figure 1A). When seeds are soaked in water, the endosperm increases in volume and becomes gelatinous (Figure 1B). Under SEM, the endosperm appears as a compact structure with sparsely distributed long-cylindrical cells (Figure 1C,D). The intercellular spaces are filled with exudates, forming a matrix in which the cells are embedded (Figure 1C,D).

**Figure 2:**
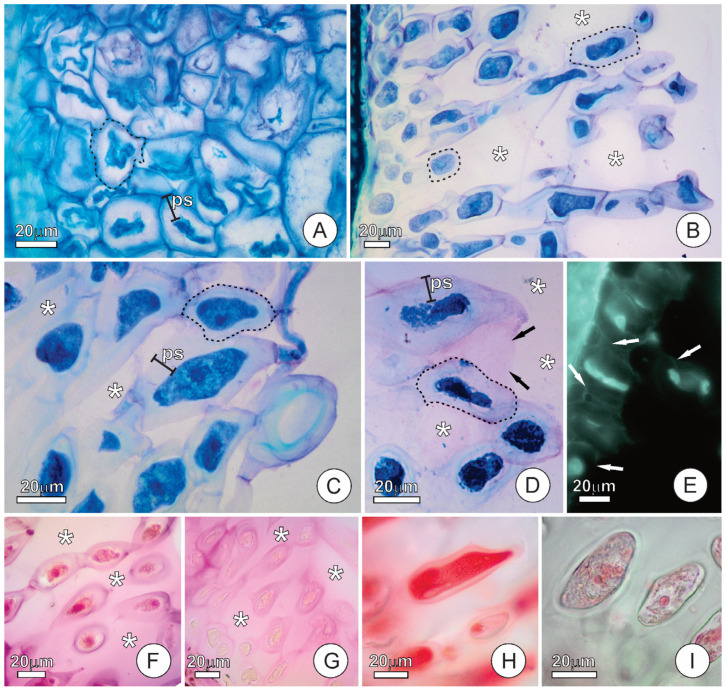
Structure of *Caesalpinia pulcherrima* endosperm at successive seed developmental stages. (**A**–**D**) Sections stained with toluidine blue. (**A**) Endosperm at stage 1; note that the endosperm is composed of juxtaposed cells showing condensed protoplasts and periplasmic spaces filled with exudates. (**B**) General view of the endosperm at stage 3, showing cells with distinct degrees of protoplast condensation and accumulation of exudates in the periplasmic space. (**C**) Detail of endosperm cells at stage 3 showing exudates filling the periplasmic spaces. (**D**) Endosperm cells at stage 4 showing condensed protoplasts and exudates filling the periplasmic spaces; note that the exudates within the intercellular space stained with toluidine blue (black arrows). (**E**–**I**) Histochemical tests of endosperm from mature seed in resin-embedded (**E**–**G**) and freehand (**H**,**I**) sections. (**E**) Calcofluor white fluoresces cellulose on cell walls in bright green (white arrows). (**F**) Ruthenium red reveals acid polysaccharides in pink staining. (**G**) Periodic acid–Schiff (PAS) highlights neutral polysaccharides in pink. (**H**) Xylidine ponceau identifies proteins in red. (**I**) Sudan Red B shows lipids in red. Asterisks mark the intercellular spaces, and the dashed black lines delimit the contour of cells. (ps = periplasmic space).

At stage 1, the endosperm of *C. pulcherrima* is a compact structure, formed by juxtaposed cells, presenting inconspicuous intercellular spaces (Figure 2A). The protoplasts are compressed, and the periplasmic spaces are filled by an amorph exudate (Figure 2A). At stages 2 and 3, endosperm cells exhibit varying degrees of protoplast compression and accumulation of exudates in the periplasmic space; some cells display fully expanded protoplasts and few exudates in the periplasmic spaces (Figure 2B), while others show compressed protoplasts and substantial accumulation of exudates in the periplasmic space (Figure 2B,C). These contrasting cellular states reflect successive rounds of protoplast compression and exudate accumulation into the periplasmic space, which occur repeatedly during stages 2 and 3. The accumulation of exudates in the periplasmic space is a dynamic process, interspersed with protoplast expansions that facilitate their release into the intercellular space. Specifically, protoplast expansion exerts pressure on the exudate, causing it to traverse the cell wall and exit the cell. Each cycle of protoplast contraction and expansion results in additional exudate being transferred into the intercellular space, thereby increasing its volume. At stage 4, the cells exhibit compressed protoplasts and accumulation of exudates in the periplasmic spaces (Figure 2D). During this stage, both protoplast compression and exudate accumulation inside the periplasmic space are more pronounced than in the earlier stages (Figure 2D). As revealed by the calcofluor white test, cellulose is detected only in the thin primary cell wall, not in the exudates accumulated in the periplasmic spaces (Figure 2E).

Additional histochemical tests revealed the presence of acidic polysaccharides in the exudates filling the periplasmic and intercellular spaces of cells in mature seeds (Figure 2F). Neutral polysaccharides were also detected in the exudates within the periplasmic and intercellular spaces (Figure 2G). Proteins and lipids were detected in the protoplast of endosperm cells (Figure 2H,I). Starch was not detected.

### 2.2. Endosperm Ultrastructure and Polysaccharides Exudation

**Figure 3:**
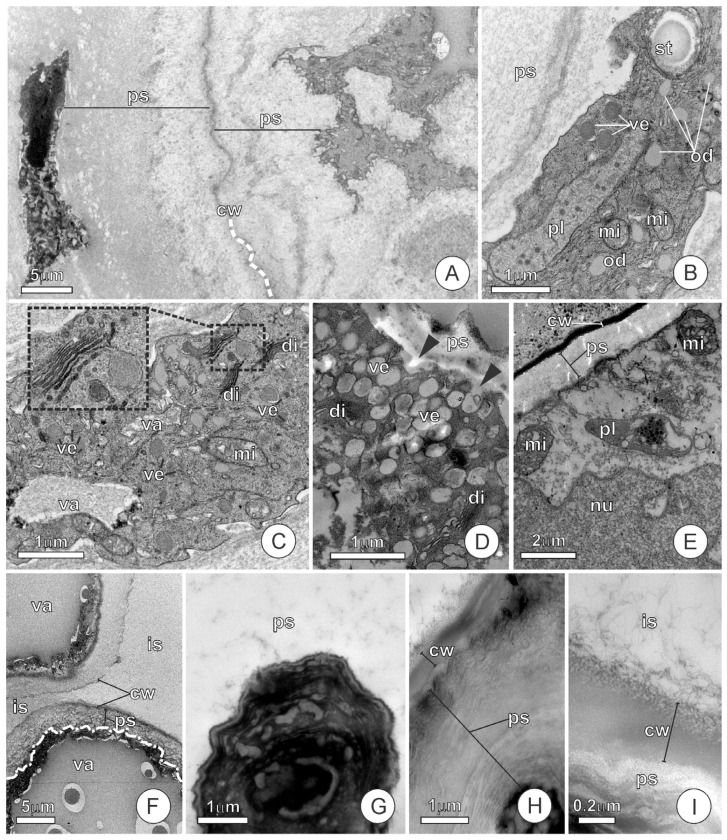
Ultrastructural aspects of the endosperm of *Caesalpinia pulcherrima* seeds at different developmental stages. (**A**–**C**) Stage 1. (**A**) General view of two endosperm cells with exudates filling their periplasmic spaces; the dashed white line indicates the cell walls. (**B**,**C**) Cells showing organelle-rich cytoplasm and periplasmic spaces filled with secretion. In the insert in C, note the vesicle sprouting from the dictyosome cisternae. (**D**,**E**) Stage 2. (**D**) Cell with vesicles scattered throughout the ground cytoplasm; note the vesicles fused with the plasma membrane (black arrowheads) that release their contents into the periplasmic space. (**E**) Detail of a cell showing organelles in the cytoplasm; note that this cell contains less secreted material in the periplasmic space, which is therefore narrower than in (**A**,**B**). (**F**) Large-vacuolated cells; note that the extra-vacuolar cytoplasm is compressed by both the large vacuole and the secretion filling the periplasmic space, appearing as a thin electron-dense layer between them. The dashed line indicates the protoplast boundaries. (**G**–**I**) Stage 3. (**G**) Detail of a cell protoplast with a lobed shape and condensed electron-dense cytoplasm. (**H**) Detail of the periplasmic space filled with a lamellar appearance secretion. (**I**) Detail of the surface of an endosperm cell, note that there are exudates in both the periplasmic space and the intercellular space, evidencing the transposition of the barrier imposed by the cell wall. (cw = cell wall; di = dictyosome; is = intercellular space; mi = mitochondria; nu = nucleus; od = oil droplet; pl = plastid; ps = periplasmic space; st = starch; va = vacuole; ve = vesicles).

At stages 1 and 2, the endosperm cells of *C. pulcherrima* show thin primary cell walls and a diversity of organelles (Figure 3A–E), including plastids, mitochondria, a well-developed Golgi apparatus, and a vacuolar system. The dictyosomes exhibit different levels of activity, producing vesicles which remain dispersed throughout the ground cytoplasm (Figure 3C,D) until they either fuse to form vacuoles or merge with the plasma membrane (Figure 3D). During stages 1 and 2, the cells contain vacuoles of varying sizes, temporarily storing material produced by the dictyosomes (Figure 3C,F); in some cases, a single large vacuole almost completely occupies the protoplast, compressing the extra-vacuolar cytoplasm to the cell boundaries (Figure 3F). The material within the vacuoles is released to the periplasmic space when the vacuolar and plasma membranes come in contact. With concomitant accumulation of exudates in the periplasmic space, the cells exhibit varying degrees of protoplast compression (Figure 3A,F). Vesicle production, vacuole formation, and exudate accumulation in the periplasmic space occur dynamically, recurring throughout stages 1 and 2.

At stage 3, the ultrastructural features of the endosperm cells become more uniform than in earlier stages. The protoplasts are compressed, exhibit a lobed shape, and contain electron-dense material, within which only electron-lucent structures resembling vacuoles remain distinguishable (Figure 3G). The polysaccharide exudates filling the periplasmic spaces acquire a condensed, lamellate appearance (Figure 3H). As in the previous stages, the cell walls remain thin, and the polysaccharides cross them, being transferred to the wide intercellular spaces (Figure 3H,I).

## 3. Discussion

As typically observed in Leguminosae [1], the endosperm of *C. pulcherrima* is hard and exhibits a vitreous appearance when dry, but becomes gelatinous and expands in volume when imbibed. This change in endosperm consistency has been associated with the hydrophilic properties of galactomannans typically found in leguminous endosperms (see [2]). This association must also apply to the polysaccharides found in the *C. pulcherrima* endosperm, previously characterized as galactomannans [4,7]. Reid and Bewley [27], Meier and Reid [2], and Buckeridge et al. [9] proposed that, due to their hydrophilicity, galactomannans also perform functions related to water regulation during seed germination, in addition to their role as storage polysaccharides. Since galactomannans account for about 30% of the weight of mature *C. pulcherrima* seeds [4], they may substantially contribute to water regulation during seed germination by creating a gelatinous enclosure that ensures uniform embryo hydration during seed imbibition and prevents seedling desiccation shortly after the emergence of the primary root. This function would be especially relevant considering that *C. pulcherrima* naturally occurs in seasonally dry environments [5,38].

During development, we observed substantial anatomical modifications in the endosperm of *C. pulcherrima*. These modifications are associated with the dynamic accumulation of exudates from endosperm cells in the periplasmic and intercellular spaces during seed development. In mature seeds, the intercellular and the periplasmic spaces are filled by these exudates. The detection of neutral polysaccharides in both compartments supports the interpretation that these exudates consist of galactomannans, which form a matrix within the endosperm. Acidic polysaccharides were also detected in the intercellular and periplasmic spaces. Although not the main components of the endosperm matrix, and not characterized as storage reserves, acid polysaccharides may contribute to water regulation during germination. These substances are typically produced by colleters [39,40,41], and, due to their hygroscopic nature, contribute to the protection of young plant tissues against desiccation. Histochemical tests further indicate the presence of proteins and lipids in the protoplasts of endosperm cells. Endosperm cells of *C. siliqua* also contain protein bodies in their cytoplasm, which are degraded during germination [31]. In this way, lipids and proteins are likely to serve as reserves and be mobilized during seed germination in *C. pulcherrima*.

The ultrastructure of the endosperm cells of *C. pulcherrima* evidences that these cells possess highly active metabolism, at least until seed physiological maturity (stage 3, the last stage we have evaluated under TEM). Features such as dense cytoplasm, conspicuous nuclei, and numerous organelles, including intense vesicle production by the Golgi apparatus in the endosperm cells, support our interpretation. The Golgi apparatus is involved in the synthesis and secretion of pectic and hemicellulosic polysaccharides [24,42], and is also associated with the secretory activity of polysaccharide-producing structures such as colleters [43,44], mucilage idioblasts [45,46,47], and trichomes [40]. Therefore, it is safe to infer that the galactomannans present in the endosperm of *C. pulcherrima* [4] are produced by the Golgi apparatus. In addition to a highly active Golgi apparatus, we also observed Golgi-derived vesicles fusing to form large vacuoles, vesicles fusing with the plasma membrane, cells with condensed protoplasts accumulating exudates in the periplasmic space, and these exudates crossing the cell wall towards intercellular spaces. Altogether, these findings provide strong evidence of the cyclic mechanism of secretion proposed by Paiva [48], in which the Golgi apparatus plays a central role. Furthermore, these findings strongly support the endosperm cells of *C. pulcherrima* as functionally secretory. It is important to emphasize that specific steps of the secretory cycle, particularly turgor-driven wall translocation, are inferred rather than directly observed in the present study. Nevertheless, even in the absence of direct evidence for these steps, it is indisputable that carbohydrates deposited in the intercellular spaces must have crossed the cell wall barrier.

Polysaccharides produced by the Golgi apparatus are transported through the cytoplasm in Golgi-derived vesicles, which can fuse to form large vacuoles. These vacuoles eventually merge with the plasma membrane, releasing their contents into the periplasmic space. Additionally, small vesicles or microvacuoles in the peripheral cytoplasm may fuse directly with the plasma membrane, also contributing to exudate release into the periplasmic space. The accumulation of exudates in the periplasmic space compresses the protoplast, leading to its condensation, as illustrated here. Driven by turgor pressure, the protoplast expands and pushes the polysaccharides through the cell wall. However, in contrast to epidermal secretory structures in which the polysaccharides are secreted to the exterior of the plant body [49], in *C. pulcherrima*, the polysaccharides are accumulated in the intercellular space in successive secretory cycles until all intercellular spaces are filled. At this stage, the protoplast enters a latent period, and the exuded polysaccharides within the intercellular spaces prevent the expansion of the protoplast and exudation of the material accumulated in the periplasmic space. Therefore, galactomannans produced in subsequent secretory cycles begin to accumulate in the periplasmic space, causing protoplast compression. At this point, the cells showed not only condensed protoplasts, but also a high degree of galactomannan condensation in the periplasmic space, as evidenced by its lamellate appearance under TEM. It becomes evident that galactomannan production remained intense throughout the complete filling of the intercellular spaces and part of the periplasmic spaces. Protoplast condensation due to carbohydrate accumulation in the periplasmic space is commonly observed in polysaccharide-secreting structures, such as mucilage idioblasts, as noted by Paiva [48]. Besides *Caesalpinia pulcherrima*, the storage of structural polysaccharides within the endosperm intercellular spaces has also been reported in *Cercis siliquastrum* [19,20]. However, *C. siliquastrum* accumulates galactoglucomannans as storage polysaccharides [36], rather than galactomannans as observed in *C. pulcherrima* [11] and other leguminous species (see [9]). To the best of our knowledge, *C. siliquastrum* is the only legume species reported to store galactoglucomannans in the endosperm and, prior to this study, was also the only one known to accumulate structural polysaccharides within the intercellular spaces.

In contrast to what was observed in *C. pulcherrima*, Meier and Reid [37] described galactomannan production in *T. foenum-graecum* as being mediated by vesicles derived from the rough endoplasmic reticulum, with no evidence of involvement of the Golgi apparatus. The authors further reported that dictyosomes are rarely observed in endosperm cells and appear to be inactive. By contrast, our observations reveal frequent and active dictyosomes, providing clear evidence of Golgi apparatus participation in galactomannan secretion. Since only two species have been investigated in the available literature, it is not possible to assess any inference about how the galactomannan production processes mediated by the rough endoplasmic reticulum and the Golgi apparatus are distributed within Leguminosae. Therefore, further studies on this topic are necessary.

The endosperm cells of *C. pulcherrima* remain alive at seed maturity (V Bonifácio-Leite, unpubl. res.), and the tissue does not possess a differentiated epidermal cell layer (aleurone layer) at its periphery. Similar features have also been reported in other leguminous species, such as *C. siliqua* [31], *C. siliquastrum* [19,20], and *Schizolobium parahyba* (Vell.) S.F.Blake [9]. In contrast, species such as *C. tetragonoloba* [33], *M. sativa*, *T. incarnatum*, and *T. foenum-graecum* [29] present the endosperm structurally divided into the peripheral aleurone layer and the storage tissue proper, in which the cells degenerate during seed development due to the lumen being almost filled with galactomannans. Meier and Reid [2] hypothesized that the aleurone layer is responsible for producing and releasing the enzymes required for the degradation of the galactomannans in the storage tissue proper. However, in the absence of an aleurone layer, this function is attributed to the living endosperm cells themselves [9], presumably the case in *C. pulcherrima*. Except for *S. virgata*, which possesses an aleurone layer and retains living endosperm at seed maturity [23], the association between the presence of an aleurone layer and a storage tissue proper composed of dead cells appears to be valid for many species from Leguminosae. Further studies on endosperm structure in additional species are essential to verify whether this association is consistent.

Several authors have referred to galactomannans as cell wall storage polysaccharides (CWSP) and described them as deposited as wall thickenings in the endosperm cells of leguminous species (see [2]). Seiler [31] described the galactomannans in the endosperm cells of *C. siliqua* as an additional cell wall layer with variable thickness and distinct fibrillar structure under the transmission electron microscope. Meier and Reid [37] analysed galactomannan production in the non-living endosperm cells of *T. foenum-graecum*. They noted that galactomannan deposition did not occur uniformly on the cell walls. Although the authors consider this galactomannan layer part of the cell wall, it is distinguishable from the primary wall under TEM, as we also observed in *C. pulcherrima*. In fact, as evidenced by the calcofluor white test, the cell walls of *C. pulcherrima* are delicate and much thinner than the periplasmic space filled with galactomannans. In contrast, pure mannans and xyloglucans are recognizable, respectively, as wall thickenings in the endosperm of several palm species [50,51,52] and in the cotyledons of some leguminous species, such as *Copaifera langsdorffii* Desf. [53] and *Hymenaea courbaril* L. [54]. Therefore, there appears to be a clear distinction in the mode of deposition of different structural reserve polysaccharides regarding their incorporation into the cell walls. While pure mannans and xyloglucans are indeed incorporated within the wall structure, galactomannans appear to form a distinct layer adjacent to it, similar to what was reported by Baldan et al. [19] and Rascio et al. [20] in *C. siliquastrum* seeds.

Thus, while the term CWSP is appropriate for genuinely wall-incorporated polysaccharides such as pure mannans and xyloglucans [50,51,52,53,54], its use requires caution and should not be generalised. Compelling evidence demonstrates that not all hemicellulose-type polysaccharides are incorporated into the cell wall. Particularly for galactomannans, it is essential to acknowledge that in at least some species, the designation CWSP is inaccurate. Although these reserves are closely apposed to the wall, they are unequivocally extra-wall in nature.

## 4. Materials and Methods

### 4.1. Plant Material and Sampling

Seeds were collected from five individuals of *Caesalpinia pulcherrima* growing in a natural environment in the municipality of Belo Horizonte (19°51′35.5″ S, 43°57′22.9″ W), Minas Gerais state, Brazil, between September 2023 and March 2024. Voucher specimens were deposited at the BHCB herbarium of the Universidade Federal de Minas Gerais (BHCB226932, BHCB227028, BHCB227082).

**Figure 4:**
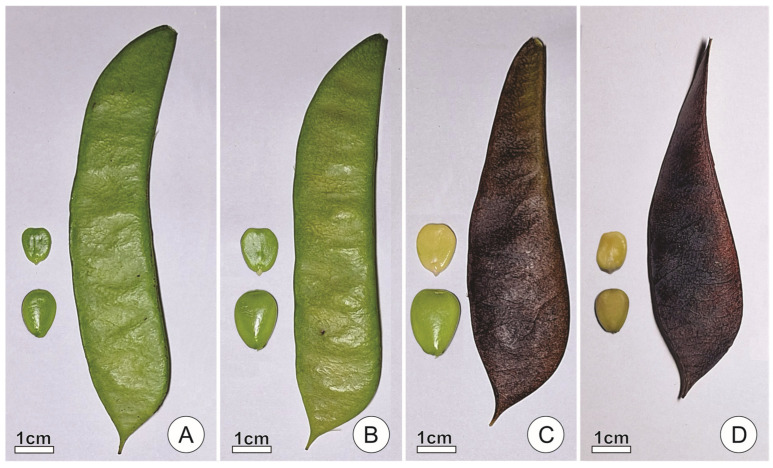
Fruits, seeds, and embryos of *Caesalpinia pulcherrima* at successive developmental stages. (**A**) Stage 1, with completely green fruits, seeds with approximately half of those at physiological maturity, and green embryos. (**B**) Stage 2, with fruits with less than 25% of their surface turned brown, seeds with a volume of circa 80% of the seeds at physiological maturity, and green embryos. (**C**) Stage 3, with fruits with more than 75% of their surface turned brown, seeds at physiological maturity, displaying the maximum volume reached during development, and cream-colored embryos. (**D**) Stage 4, with entirely brown, dry, dehiscent fruits, and hard, desiccated, fully mature seeds.

Endosperm samples for microscopy analyses were collected from seeds at four developmental stages, which were defined based on morphological characteristics of the fruits and seeds (Figure 4). Seeds at stage 1 were obtained from completely green fruits and had approximately half the volume of seeds at physiological maturity, containing green embryos (Figure 4A). Seeds at stage 2 obtained from fruits with less than 25% of their surface turned brown and were near physiological maturity, reaching about 80% of their maximum volume and still containing green embryos (Figure 4B). Seeds at stage 3 were obtained from fruits with more than 75% of their surface turned brown and were at physiological maturity, displaying the maximum volume reached during development and having cream-colored embryos (Figure 4C). Finally, seeds at stage 4 were obtained from entirely brown, dry, dehiscent fruits; seeds were fully mature, hard, and desiccated (Figure 4D).

### 4.2. Light Microscopy and Histochemistry

For light microscopy, endosperm samples from seeds at the four stages were fixed in formalin:acetic acid:ethanol 85% (1:1:18 *v*/*v*) under slight vacuum for 5 min and then stored for 48 h in the fixative solution. During preliminary tests, the use of aqueous fixatives or the conventional formalin:acetic acid:ethanol 50% [55] led to the gelatinisation of galactomannan reserves. Therefore, the lower-water-content fixative minimised artifacts caused by the expansion of carbohydrate reserves. The samples were subsequently dehydrated in an ascending ethanol series and embedded in 2-hydroxyethyl-methacrylate (Leica Microsystems Inc., Heidelberg, Germany), according to Paiva et al. [56]. Sections (10 μm thick) were obtained using a rotary microtome (Hyrax M40, Carl Zeiss Mikroskopie, Jena, Germany), stained with 0.05% toluidine blue, pH 4.7 in acetate buffer ([57], modified), and observed under an Olympus CX41 microscope (Olympus Scientific Solutions, Waltham, MA, USA). Images were captured using a digital camera (TV0.5XC-3, Olympus Scientific Solutions, Waltham, MA, USA).

For histochemical tests, mature seeds were imbibed in water for 10 min. Freehand sections of the endosperm were obtained with a razor blade. Both freehand and embedded sections of endosperm from mature seeds were submitted to the following tests: ruthenium red aqueous solution (0.002%) for acidic polysaccharides [55], periodic acid/Schiff’s reagent (PAS) for neutral polysaccharides [58], lugol solution for starch [55], sudan red B for total lipids ([59], modified) and xylidine ponceau for proteins [60]. The metachromasy of toluidine blue was also employed to recognize acidic polysaccharides as initially proposed by O’Brien et al. [57]. According to these authors, the metachromasy of toluidine blue allows the identification of various cell types and cell wall compositions. Primary walls and middle lamellae assume reddish-purple tones in toluidine blue due to the presence of acidic polysaccharides (mainly pectin) in their composition [61].

Additionally, the histochemical test with calcofluor white for cellulose detection was also employed [62]. Calcofluor white (CFW; Sigma-Aldrich PTY Ltd., St. Louis, MO, USA, product number 18909) was used following the manufacturer’s protocol for fluorescence staining. The material was observed under a Leica DM2500 fluorescence microscope (Leica Microsystems, Wetzlar, Germany) coupled to a CCD Micromax camera (12 bits, Leica DFC345FX).

### 4.3. Electron Microscopy

For transmission electron microscopy (TEM), samples (approx. 1 mm^3^) of endosperm from seeds at the stages 1, 2 and 3 were fixed in Karnovsky fixative (pH 7.2 in 0.1 M phosphate buffer; modified from Karnovsky [63]) under slight vacuum for 24 h, post-fixed in 1% osmium tetroxide for 2 h, dehydrated in an ascending acetone series, and embedded in epoxy resin [64]. Ultrathin sections were obtained using an ultramicrotome (UC6, Leica, Wetzlar, Germany) and contrasted with uranyl acetate [65] and lead citrate [66]. The sections were observed using a transmission electron microscope Tecnai G2–Spirit (Philips/FEI, Eindhoven, The Netherlands).

For scanning electron microscopy (SEM), the median section of seeds at stage 4 (mature seeds) was fractured with a screwdriver. The fractured surfaces of the samples were sputter-coated (10 nm) with a palladium–gold alloy using an SCD030 coater (Bal-Tec, Balzers, Liechtenstein) and observed under a scanning electron microscope Quanta 200 (FEI Company, Eindhoven, The Netherlands).

## 5. Conclusions

The endosperm of *Caesalpinia pulcherrima* is formed by functionally secretory cells that produce galactomannans like the cells of other polysaccharide-secreting structures, such as colleters, mucilaginous idioblasts, and glandular trichomes. During seed development, the secretory process leads to structural modifications in the endosperm, which are related to cycles of polysaccharide accumulation in the periplasmic space, followed by protoplast expansion. This process leads to the translocation of polysaccharides across the cell wall into the intercellular space. In mature seeds, galactomannans occupy the periplasmic and intercellular spaces of the endosperm, and they are not incorporated into the cell walls, as is frequently described in the literature for leguminous seeds. Galactomannans cause the mature seeds of *C. pulcherrima* to exhibit the typical characteristics of the endosperm of Leguminosae: hard and vitreous in appearance when dry, but gelatinous and expanded when imbibed. The hydrophilic nature of galactomannans facilitates seed imbibition, providing a reserve and moisture source during germination. Although information on anatomy and galactomannan production in the endosperm of legumes is scarce in the literature, the comparison of *C. pulcherrima* with the available data revealed that the Leguminosae endosperm appears to exhibit great anatomical diversity and variation in the sites of galactomannan accumulation. Further studies focusing on endosperm development across a broader range of leguminous species are needed to address substantial knowledge gaps in galactomannan synthesis and in the anatomy and histology of legume endosperm. Our results, together with a substantial body of supporting literature, indicate that the term CWSP is appropriate for genuinely wall-incorporated polysaccharides. However, not all polysaccharides located outside the protoplasts are structurally incorporated into the wall and, therefore, cannot be considered part of it. In the case of galactomannans, it is imperative to recognize that in *C. pulcherrima*, and at least in *C. siliquastrum* [19,20], the designation CWSP is inaccurate.

## Data Availability

The original contributions presented in this study are included in the article. Further inquiries can be directed to the corresponding author.

## Abbreviations

The following abbreviations are used in this manuscript:

CWSP	Cell wall storage polysaccharides
TEM	Transmission electron microscopy
